# Increased suicide risk of psychiatric patients following the recent utilization of health care services: results from a nationwide cohort study in South Korea

**DOI:** 10.3389/fpubh.2023.1118135

**Published:** 2023-05-30

**Authors:** Ju-Mi Lee, Junhee Lee, Jiseun Lim, Soonjoo Park, Myung Ki, Jiwon Kang

**Affiliations:** ^1^Department of Preventive Medicine, College of Medicine, Eulji University, Daejeon, Republic of Korea; ^2^Department of Preventive Medicine, School of Medicine, Chungnam National University, Daejeon, Republic of Korea; ^3^Department of Psychiatry, Uijeongbu Eulji Medical Center, Eulji University, Gyeonggi, Republic of Korea; ^4^College of Nursing, Konyang University, Daejeon, Republic of Korea; ^5^Department of Preventive Medicine, College of Medicine, Korea University, Seoul, Republic of Korea

**Keywords:** suicde, health care service, psychiatric admission, psychiatric outpatient, psychiatric inpatients, South Korea

## Abstract

**Purpose:**

This study aimed to examine whether and to what degree the suicide risk of psychiatric patients is associated with psychiatric and non-psychiatric health service utilization.

**Methods:**

We selected incident psychiatric patients, including schizophrenia, bipolar disorders, borderline personality disorder, depressive disorders, other affective disorders, and post-traumatic stress disorder patients, in 2007–2010 and followed them up until 2017 based on the data linkage between the Korean National Health Insurance and National Death Registry. We analyzed the time-dependent association between suicide and four types of health service (psychiatric vs. non-psychiatric and outpatient vs. inpatient) utilization using a time-dependent Cox regression.

**Results:**

The suicide risk of psychiatric patients was significantly increased with recent psychiatric and non-psychiatric admission and psychiatric outpatient visits. The adjusted suicide hazard ratios for recent outpatient visits were similar to or even higher than those for recent psychiatric admission. The adjusted suicide hazard ratios of schizophrenia patients for psychiatric admission, psychiatric outpatient visits, and non-psychiatric admission within the recent 6 months were 2.34 (95% confidence interval [CI]: 2.12–2.58, *p* < 0.001), 2.96 (95% CI: 2.65–3.30, *p* < 0.001), and 1.55 (95% CI: 1.39–1.74, *p* < 0.001), respectively. Suicide risk was not associated with recent non-psychiatric outpatient visits in patients, except for the depressive disorders group showing a negative association.

**Conclusion:**

Our results highlight the priority of suicide prevention for psychiatric patients in the clinical setting. Additionally, our results warrant the precaution against increased suicide risk of psychiatric patients after psychiatric and non-psychiatric discharge.

## 1. Introduction

Suicide is a critical global public health concern, accounting for 1.3% of worldwide mortality, with an estimation of 703,000 death in 2019 (1). Certain Asian regions, such as South-East Asia, have higher suicide rates (10.2 per 100,000) than the global average (9.0 per 100,000) (2). South Korea (hereafter referred to as Korea) has shown the highest suicide rate of all the Organization for Economic Co-operation and Development (OECD) countries since 2003, ranging from 23.8 to 35.3 suicide deaths per 100,000 persons in South Korea (3). Nationwide Korean studies have observed that male sex, old age, low income, and unmarried or divorced status were associated with higher suicide risk among the general population (4–8). Psychiatric patients in Korea have also shown a markedly higher suicide rate than that of the general population in Korea (9).

Worldwide, many victims of suicide have a mental illness at the time of death (10), and psychiatric patients show significantly higher suicide rates than the general population, similar to the Korean statistics (11). Therefore, monitoring and managing the suicide risk of psychiatric patients is one of the instrumental suicide prevention strategies (12). At this point, knowing when the risk of suicide increases in the clinical course of a psychiatric patient can help clinicians actively find out about patients’ suicide ideation and intervene in a timely fashion.

Although there is no single point at which the risk of suicide in psychiatric patients peaks, two periods, that is, the acute phase of psychiatric illness and the time after inpatient discharge, are considered high-risk periods for suicide (11). Numerous studies, including systematic reviews, have consistently shown that the period after psychiatric inpatient discharge is a prominently vulnerable time to suicide. An inpatient cohort study found that patients with depressive disorders (DD) showed a 13.0 times higher suicide rate during the first 90 days after psychiatric discharge compared to the group with no psychiatric disorders (13). Regarding outpatient visits, over 80% and around 25% of suicide victims used non-mental and mental health care services, respectively, within the 1 year before suicide in several previous studies (14). Still, there is no evidence of whether suicide risk changes with psychiatric or non-psychiatric outpatient visits among psychiatric patients, to our best knowledge.

To fill the knowledge gap concerning the association between the suicide risk of psychiatric patients and their health care service utilization, several aspects warrant further investigation. First, considering the innate fragility of psychiatric patients regarding suicidality, the impact on suicide risk needs to be separated into health care service utilization and the morbidity of psychiatric disorders. The cause of the extremely high suicide risk after discharge from psychiatric care reported in previous studies can be partitioned into risk from the psychiatric disorder itself and risk from the extent of time, and there is a lack of understanding of the changes in suicide risk associated with psychiatric admission itself. Second, a comprehensive range of health care services, including non-psychiatric inpatient care (NI), and psychiatric and non-psychiatric outpatient care (PO and NO), besides psychiatric inpatient care (PI), needs to be examined. Second, it is necessary to examine a comprehensive range of medical services beyond psychiatric inpatient treatment (PI), including non-psychiatric inpatient treatment (NI) and psychiatric and non-psychiatric outpatient treatment (PO and NO, respectively). Evidence supports that physical illness can play an important role in suicide, suggesting that suicide risk can also increase after discharge from NI (15). Additionally, psychiatric patients can express their mental distress as well as suicide ideation with various medically unexplained somatic complaints or pain, implying a positive association between NO visits and suicide risk (16). Third, the association between suicide and health care service utilization needs to be examined for each psychiatric disorder, given the possibility that the health care service utilization by patients with suicide ideation as a help-seeking behavior and the effectiveness of health care services can vary depending on the affected psychiatric disorder. Analyzing associations across disorders can produce detailed evidence for the tailored suicide prevention strategy for each psychiatric disorder.

To address these issues, we conducted this study to examine how suicide hazard is associated with the various health care service utilizations, including psychiatric versus non-psychiatric and inpatient versus outpatient care, by incident patients across psychiatric disorders. The time-dependent suicide hazard according to health care service utilization within the recent 1 month and recent 6 months was examined using nationally representative cohort data. We aimed to contribute to the development of suicide prevention strategies for psychiatric patients, particularly for Korean psychiatric patients, for whom the suicide rate is very high.

## 2. Materials and methods

### 2.1. Study population

This study was executed with a retrospective cohort design using the National Health Information Database (NHID) (NHIS-2019-1-009) provided by Korean National Health Insurance Service (NHIS), a public database containing nationwide medical claim information from the compulsory health care insurance system. The study population was six groups of patients who were over 15 years old and newly diagnosed with schizophrenia (SZ; Korean Standard Classification of Disease [KCD] code: F20), bipolar disorders (BD; F31), borderline personality disorder (BPD; F60.3), DD (F32, F33), other affective disorders (OAD; F30, F34, F38, F39), and post-traumatic stress disorder (PTSD; F43.1).

We selected those who used medical services more than twice for a mental illness in 2007–2010 and excluded those already diagnosed with the same illness in 2002–2006 to extract only incident psychiatric patients. The final numbers of participants were 102,540 for SZ; 96,366 for BD; 6,476 for BPD; 1,235,465 for DD; 376,621 for OAD; and 12,973 for PTSD. Patients diagnosed with more than one psychiatric disorder were included in multiple groups.

The Institutional Review Board of Eulji University approved this study (EU2019-25). The committee waived the requirement for written informed consent as this study used secondary data with no personal information.

### 2.2. Measurements

The outcome event was a suicide death identified based on KCD codes X60-X84, using the National Statistics Organization database. The independent variables were time-dependent PI, PO, NI, and NO within the previous month and the previous 6 months. Covariates included age, sex, income level, residence area, comorbidity score using the Charlson comorbidity index (CCI), and comorbidity with other psychiatric disorders. Age was classified into seven categories using 10-year intervals (15–24, 25–34, 35–44, 45–54, 55–64, 65–74, and 75+). Income level was classified by whether a participant was eligible for medical aid or national health insurance. Then, the national health insurance group was divided into low (≤17.41 USD), middle-low (≤33.42 USD), middle-high (≤62.66 USD), and high (>62.66 USD) categories based on the patient’s premium amount. The residence area was categorized into metropolitan and rural areas. CCI scores were calculated using Quan’s method based on the KCD code and grouped into three categories: 0, 1–6, and, ≥7 (17). The number of comorbid psychiatric disorders was counted based on 11 categories of psychiatric disorders (described in Supplementary Material) and collapsed into 0 (no comorbidity), 1–2, and, ≥3. Comorbidity measures, including CCI and comorbid psychiatric disorders, were evaluated from 2 years before to 1 year after the first diagnosis of a psychiatric disorder.

### 2.3. Statistical analysis

The first diagnosis was taken as the index time, and the follow-up endpoint was the date of death or December 31, 2017. Crude and characteristic-specific suicide rates per 100,000 person-years were calculated using a generalized linear model based on the Poisson distribution (Proc Genmod statement in SAS 9.4, SAS Institute, Cary, NC, United States). The associations of suicide hazards with health care service utilization were examined with time-dependent Cox proportional hazard models using the programming statement in SAS. The time-dependent variables *PI(i)*, *PO(i)*, *NI(i)*, *NO(i)*, and *SA(i)* were created, respectively, for PI, PO, NI, NO, and any kind of health care services utilized due to suicide attempts in the _i__th_ month and were included in the Cox proportional hazard model as follows:


$$\begin{array}{c} h\left(i\right)=h_{o}\left(i\right)\\ \times \exp\left[\beta_{1}PI\left(i\right)+\beta_{2}PO\left(i\right)+\beta_{3}NI\left(i\right)+\beta_{4}NO\left(i\right)+\beta_{5}SA\left(i\right)+\beta_{c}C\right] \end{array}$$

where *h(i)* is the individual suicide hazard at time *i*, *h_o_*(*i*) is the baseline suicide hazard at time *i*, *β*_1_−*β*_5_ are coefficients of the time-dependent variables, *β*_c_ is the vector of covariate coefficients, and *C* is the vector of covariates.

More explanations are presented for the time-dependent variables and the time-dependent Cox model in the Supplementary Material. There was little difference in the analysis results considering the competing risk from the primary results, so we presented the results without considering the competing risk. Differences were considered significant at *p* < 0.05. Data processing and statistical analyzes were performed using SAS 9.4.

## 3. Results

### 3.1. Baseline characteristics of the cohort

The SCZ and BD groups showed a relatively even age distribution, whereas patients with BPD accounted for approximately 60% of the patients under 35 years of age (Table 1). Patients with DD or OAD and PTSD patients had a large population in late adulthood and early adulthood, respectively. Male and female SCZ patients were similar, and many SCZ patients were medical aid recipients (23.5%), but other patient groups had more female patients and higher household income. Approximately 50% of psychiatric patients had a CCI score of 1–6 and one or two psychiatric comorbidities.

**Table 1:** The baseline characteristics among the cohort of schizophrenia, bipolar disorders, borderline personality disorder, depressive disorders, other affective disorders, and post-traumatic stress disorder groups.

Characteristics	Schizophrenia		Bipolar disorders		Borderline personality disorder		Depressive disorders		Other affective disorders		Post-traumatic stress disorder	
	*N*	%	*N*	%	*N*	%	*N*	%	*N*	%	*N*	%
Age												
15–24	12,426	12.1	12,941	13.4	2059	31.8	87,673	7.1	22,295	5.9	2,407	18.6
25–34	15,095	14.7	14,613	15.2	1872	28.9	119,729	9.7	34,729	9.2	2,136	16.5
35–44	18,921	18.5	15,989	16.6	1,086	16.8	174,947	14.2	55,904	14.8	2,459	19.0
45–54	19,278	18.8	15,998	16.6	644	9.9	254,197	20.6	82,649	21.9	3,042	23.4
55–64	12,127	11.8	11,485	11.9	314	4.8	216,105	17.5	71,305	18.9	1,696	13.1
65–74	11,939	11.6	13,035	13.5	287	4.4	233,813	18.9	73,348	19.5	895	6.9
75+	12,754	12.4	12,275	12.7	214	3.3	149,001	12.1	36,391	9.7	338	2.6
Sex												
Males	48,951	47.7	39,817	41.3	2,564	39.6	437,786	35.4	129,483	34.4	4,752	36.6
Females	53,589	52.3	56,519	58.7	3,912	60.4	797,679	64.6	247,138	65.6	8,221	63.4
Income												
Medical aid	24,110	23.5	10,860	11.3	665	10.3	123,572	10.0	30,479	8.1	1,175	9.1
Low	17,074	16.7	15,183	15.8	1,257	19.4	206,394	16.7	62,968	16.7	2,512	19.4
Middle-low	16,424	16.0	16,914	17.6	1,357	21.0	230,929	18.7	71,362	18.9	2,800	21.6
Middle-high	17,953	17.5	20,321	21.1	1,280	19.8	275,468	22.3	85,744	22.8	2,909	22.4
High	26,979	26.3	33,058	34.3	1917	29.6	399,102	32.3	126,068	33.5	3,577	27.6
Region												
Seoul/metropolitan cities	43,031	42.0	41,405	43.0	2,929	45.2	486,920	39.4	144,127	38.3	5,607	43.2
Others	59,489	58.0	54,931	57.0	3,547	54.8	748,537	60.6	232,494	61.7	7,366	56.8
Charlson comorbidity index score												
0	28,784	28.1	19,632	20.4	2,464	38.0	254,882	20.6	76,197	20.2	4,553	35.1
1 ~ 6	63,958	62.4	66,956	69.5	3,883	60.0	913,268	73.9	284,611	75.6	8,129	62.7
≥7	9,798	9.6	9,748	10.1	129	2.0	67,315	5.4	15,813	4.2	291	2.2
Psychiatric comorbidity												
0	17,830	17.4	11,491	11.9	657	10.2	460,268	37.3	139,726	37.1	2,824	21.8
1 ~ 2	53,284	52.0	61,283	63.6	3,241	50.2	665,842	53.9	181,983	48.3	6,907	53.2
≥3	31,426	30.6	23,562	24.5	2,560	39.6	109,355	8.9	54,912	14.6	3,242	25.0

### 3.2. Suicide rate of six psychiatric patients

**Figure 1:**
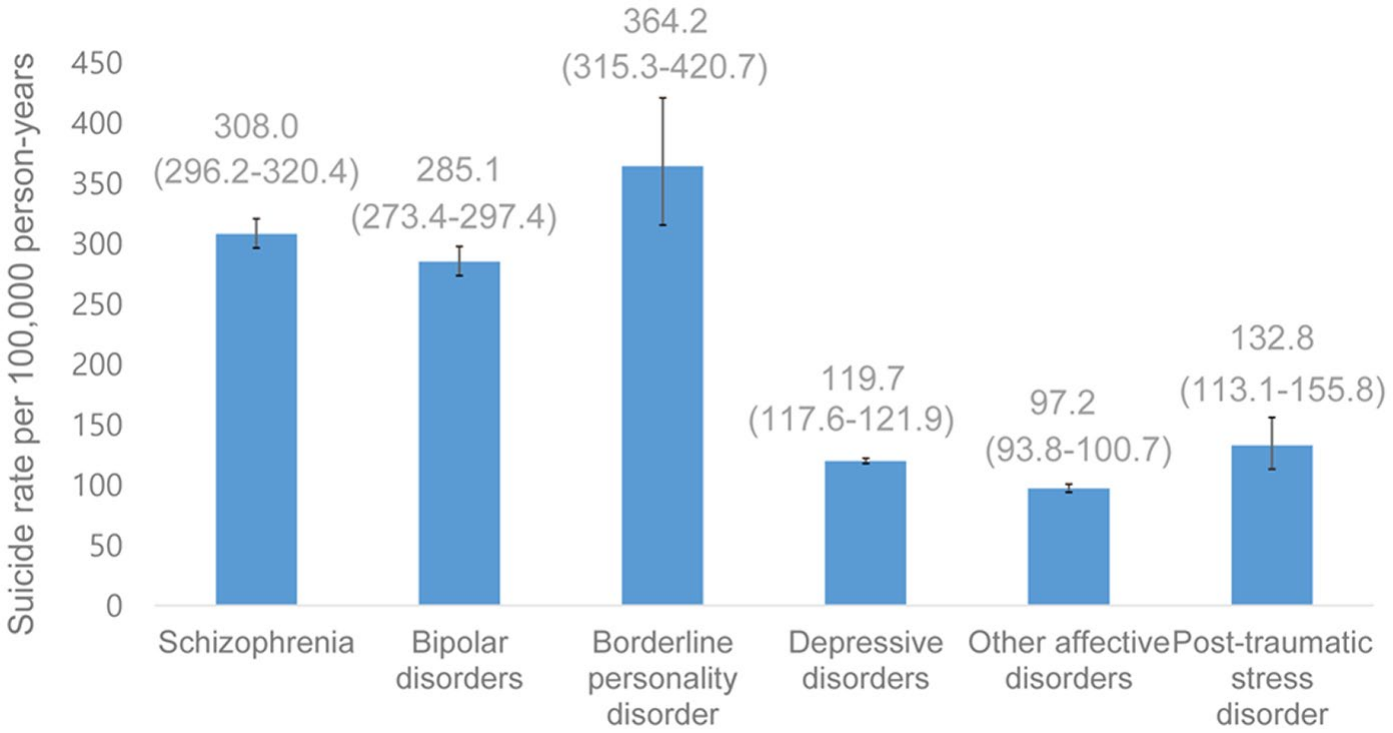
Suicide rate per 100,000 person-years (95% confidence interval) among schizophrenia, bipolar disorders, borderline personality disorder, depressive disorders, other affective disorders, and post-traumatic stress disorder patients.

The suicide rate was the highest among patients with BPD (364.2 per 100,000), followed by SZ (308.0 per 100,000) and BD (285.1 per 100,000) (Figure 1). The suicide rate was relatively low in patients with OAD (97.2 per 100,000), DD (119.7 per 100,000), and PTSD (132.8 per 100,000).

### 3.3. Suicide hazard associated with health care service utilization

The crude suicide hazard ratios (HRs) in relation to recent PI, NI, and PO were consistently positive, while the sizes of association were various across six disorder groups (Table 2). The suicide HRs for PI within the previous 6 months were 10.81 (95% CI: 9.57–12.22, *p* < 0.001), 6.63 (95% CI: 3.95–11.11, *p* < 0.001), 3.21 (95% CI: 2.89–3.56, *p* < 0.001), and 2.09 (95% CI: 1.91–2.29, *p* < 0.001) in OAD, PTSD, BD, and SZ groups, respectively. In general, the association between recent NO visits and suicide risk was not statistically significant.

**Table 2:** Crude suicide hazard ratio for recent utilization of health care services.

Health care services	Schizophrenia		Bipolar disorders		Borderline personality disorder		Depressive disorders		Other affective disorders		Post-traumatic stress disorder	
	HR	*p*	HR	*p*	HR	*p*	HR	*p*	HR	*p*	HR	*p*
PI in the previous month	1.38 (1.24–1.54)	<0.001	2.29 (2.01–2.62)	<0.001	1.76 (1.05–2.95)	0.031	2.62 (1.87–3.67)	<0.001	9.12 (7.77–10.70)	<0.001	6.35 (3.49–11.57)	<0.001
PO in the previous month	1.89 (1.74–2.04)	<0.001	2.41 (2.21–2.63)	<0.001	3.67 (2.74–4.91)	<0.001	1.68 (1.45–1.94)	<0.001	5.55 (5.16–5.96)	<0.001	4.49 (3.22–6.26)	<0.001
NI in the previous month	1.20 (1.05–1.38)	0.009	1.62 (1.40–1.88)	<0.001	2.42 (1.49–3.93)	<0.001	1.83 (1.29–2.59)	<0.001	4.14 (3.76–4.57)	<0.001	2.60 (1.52–4.44)	<0.001
NO in the previous month	1.04 (0.96–1.13)	0.342	0.98 (0.90–1.06)	0.554	1.48 (1.11–1.97)	0.008	1.05 (0.90–1.22)	0.527	1.16 (1.08–1.26)	<0.001	1.02 (0.74–1.41)	0.904
PI in the previous 6 months	2.09 (1.91–2.29)	<0.001	3.21 (2.89–3.56)	<0.001	3.02 (2.09–4.36)	<0.001	4.07 (3.27–5.08)	<0.001	10.81 (9.57–12.22)	<0.001	6.63 (3.95–11.11)	<0.001
PO in the previous 6 months	2.84 (2.56–3.16)	<0.001	2.91 (2.61–3.24)	<0.001	5.04 (3.53–7.20)	<0.001	2.26 (2.02–2.52)	<0.001	5.97 (5.53–6.44)	<0.001	5.45 (3.76–7.90)	<0.001
NI in the previous 6 months	1.40 (1.26–1.56)	<0.001	1.73 (1.55–1.92)	<0.001	2.20 (1.56–3.11)	<0.001	2.30 (1.87–2.83)	<0.001	3.05 (2.82–3.30)	<0.001	2.86 (1.96–4.17)	<0.001
NO in the previous 6 months	1.11 (1.01–1.22)	0.031	1.06 (0.94–1.20)	0.349	1.61 (1.01–2.57)	0.045	1.52 (1.37–1.70)	<0.001	1.02 (0.88–1.17)	0.830	1.24 (0.71–2.16)	0.448

PI, psychiatric inpatient care; PO, psychiatric outpatient care; NI, non-psychiatric inpatient care; NO, non-psychiatric outpatient care.

**Figure 2:**
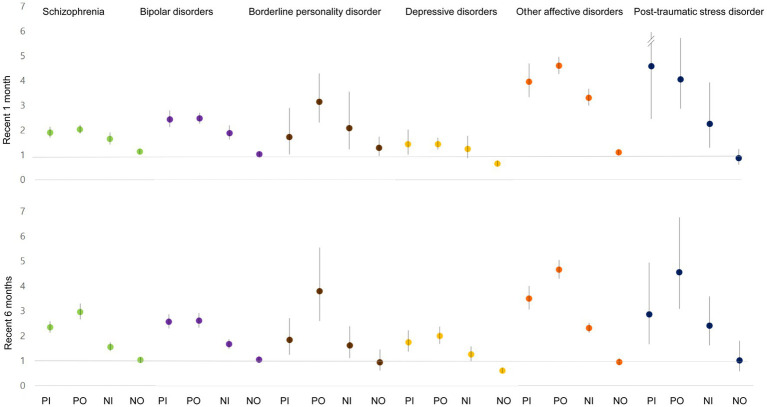
Suicide hazard ratio and 95% confidence interval for recent utilization of health care services after adjustment for age, sex, income, residence area, comorbidity with physical and psychiatric disorders, and the utilization of other health care services. PI, psychiatric inpatient care; PO, psychiatric outpatient care; NI, non-psychiatric inpatient care; NO, non-psychiatric outpatient care.

After adjustment for age, sex, income, residence area, comorbidity with physical and psychiatric illnesses, and the utilization of other health care services, the pattern of association across service types and disorders was maintained, but the size of associations decreased (Figure 2). The adjusted suicide HR was similar between PI and PO within the previous month, while PO within the recent 6 months showed a stronger association with suicide than PI among the SZ, BPD, OAD, and PTSD groups. The adjusted suicide HRs for PI, PO, and NI within the recent 6 months were 2.34 (95% CI: 2.12–2.58, *p* < 0.001), 2.96 (95% CI: 2.65–3.30, *p* < 0.001), and 1.55 (95% CI: 1.39–1.74, *p* < 0.001) in SZ patients, respectively (Supplementary Table S1). The null association was found between NO and suicide risk except in patients with DD, who showed a significantly negative association: adjusted HRs for NO visits in the previous one and 6 months were 0.64 (95% CI: 0.54–0.77, *p* < 0.001) and 0.62 (95% CI: 0.53–0.74, *p* < 0.001), respectively.

## 4. Discussion

In this nationally representative cohort study, suicide hazard was positively associated with recent psychiatric inpatient (PI), psychiatric outpatient (PO), and non-psychiatric inpatient care (NI) consistently across patients with six psychiatric disorders. On the other hand, a null association was found between recent non-psychiatric outpatient (NO) and suicide among patients except for DD group, which showed a negative association. Contrary to expectations, the association of recent PO with suicide was similar to or more prominent than that of recent PI with suicide risk after adjustment.

In the current study, increased suicide risk was significantly associated with PI, in line with previous studies reporting elevated suicide risk after discharge. However, the size of the association in our research was small compared to previous results. The adjusted suicide HR was 8.9 during 90 days after discharge among patients with SZ in a previous study (13), while the adjusted suicide HR was 1.84 for PI within the recent 6 months among patients with SZ in the current study. This may be because the reference group of the previous research was the general population, while we compared suicide hazards associated with recent PI among patients with psychiatric disorders. This suggests that the exceptionally high suicide risk after psychiatric discharge reported in previous studies could have been partially derived from the intrinsic higher suicide risk of psychiatric patients compared to that of the general population.

The previous researches have demonstrated that one of the most high-risk periods for suicide is the time following discharge from a psychiatric ward (13, 18–24). For instance, previous studies using an American sample or Swedish national cohort reported that the suicide hazard was greatest following discharge from inpatient care (22, 25). Another study using a Canadian sample reported a higher suicide hazard among patients with SZ following PI versus PO (24). In the current study, the positive association between recent PI and suicide remarkably decreased after adjustment, resulting in PO within the recent 6 months being positively associated with suicide hazards to a similar or even higher degree compared to PI. This implies that the high suicide risk associated with recent PI was partially derived from the confounders indicating the severity of disorders, such as psychiatric comorbidity. Therefore, if we assume an equivalent severity, recent PO of psychiatric patients can be as significant of an indicator of suicide risk as recent PI. In addition, this result might imply that a major proportion of psychiatric outpatients with suicide ideation may commit suicide before having the chance to be admitted. Our results highlight the prioritization of suicide prevention strategies for psychiatric patients in clinical settings, including the active monitoring and timely intervention of suicide risk. Additionally, the positive association between suicide and recent non-psychiatric admission found in this study supports the need to keep an eye on patients during the period following non-psychiatric discharge to monitor and manage their suicide risk if they received psychiatric diagnoses. To prevent suicide in those high-risk periods, clinicians in primary health care services in Korea may implement more active and regular assessments of suicidal risk after PI and NO and during regular PO visits, using relevant instruments, such as the Columbia Suicide Severity Rating Scale (23), based on a therapeutic and empathic rapport (26) for psychiatric patients.

Notably, NO was not significantly associated with suicide hazards despite several previous results reporting increased contact with general practitioners and increased visits for physical symptoms before suicide (27, 28). Another recent study using nationally representative Korean cohort data also reported that among those who completed suicide, utilization of non-psychiatric health care was more common than that of psychiatric health care prior to suicide (29). A study using a Swedish national cohort also reported that suicide among patients with drug use disorders was often preceded by NO (30), and a Danish study reported an increased frequency of encounters with general practitioners before suicide death (31). This discrepancy in the results of studies may be derived from the difference in study populations: previous descriptive studies have observed the general population, while the current study examined psychiatric patients. In this study, the association became weaker after adjustment, indicating that the increased pattern of NO before suicide in previous studies can be partly explained by the confounding effects of covariates and the utilization of other health care services. In sum, our results suggest that NO is not associated with successive suicide risk after considering confounders.

On the other hand, patients with DD showed significantly less utilization of non-psychiatric clinics ahead of suicide, suggesting that because of their depressive symptoms, such as avolition, they may show less help-seeking behavior via non-psychiatric clinics, except for critical health care service utilization, such as hospital admissions or psychiatric outpatient visits. Our results suggest that collaborating with psychiatric and non-psychiatric health care practitioners to monitor suicide risk might be an effective suicide prevention strategy for patients with DD who also have chronic physical illnesses.

In this study, the positive associations between health care service utilization and suicide risk were prominent among patients with OAD and PTSD. An interpretation of this result could be that these patients demonstrate more help-seeking behaviors in acute conditions than patients with other disorders. Because the random error of association was substantial in the PTSD group, further studies are necessary to determine whether this pattern is consistently found. The OAD group included heterogeneous and unspecific disorders (unspecified manic episodes, persistent mood disorders, and other or unspecified mood disorders), making it difficult to interpret the more prominent association observed in this group. More work is needed to understand the patterns and mechanisms of the relationships between health care service utilization and suicide risk among patients with OAD and PTSD.

The present study has some limitations derived from the methodology. First, psychiatric disorders were measured based on the KCD code recorded for the national health insurance claim; they were not based on screening or diagnostic instruments, such as structured questionnaires. Thus, diagnostic accuracy cannot be fully guaranteed. Second, to estimate the effect of time-dependent health care service utilization on suicide hazards, we divided the follow-up period into monthly intervals. We counted health care service utilization in the previous month, not including health care service utilization in the month when the suicide happened because a person who committed suicide cannot use medical services. This approach could underestimate the association between suicide hazards and recent health care service utilization. Third, although the study participants had comorbid psychiatric disorders, the main comorbid disorder of each psychiatric patient group was not identified, which limited our understanding of the characteristics of each patient group.

Despite these shortcomings, this is the first epidemiological study to evaluate the association of suicide hazards with four types of health care service utilization among incident psychiatric patients. By analyzing time-dependent associations, not comparing the suicide risks of psychiatric patients with those of the general population, we could estimate the changes in suicide hazards associated with health care service utilization during the clinical course of psychiatric patients.

The World Health Organization (WHO) has proposed that suicide prevention strategies should be multisectoral, including the health care sector, and have clear objectives, indicators, timelines, milestones, and action plans specific to each country; thus, we suggest further investigation of the association between suicide and health care utilization in each country to inform the construction of nationally tailored suicide prevention strategies and to provide hints for identifying vulnerable populations (32). For countries with high suicide rates, like Korea, we suggest that suicide prevention strategies should also be implemented in NI care settings.

## 5. Conclusion

In conclusion, we found consistently positive suicide HRs across the recent utilization of PI, PO, and NI among the psychiatric patient groups, while recent NO showed a null association with suicide hazard. The current study revealed a similar or even higher suicide risk with recent PO than with recent PI, highlighting the need for improved suicide risk assessment and the priority of awareness of the increased suicide risk of psychiatric patients who have received PO and the timely intervention that must be provided for these patients.

## Data availability statement

Publicly available datasets were analyzed in this study. This data can be found here: The Korean National Health Information Database is an open dataset after submitting the proposal through the Korean National Health Insurance Sharing system. The website address is as follows: https://nhiss.nhis.or.kr/bd/ab/bdaba021eng.do.

## Ethics statement

The studies involving human participants were reviewed and approved by Institutional Review Board of Eulji University. Written informed consent to participate in this study was provided by the participants’ legal guardian/next of kin.

## Author contributions

JiL conceptualized the study. JiL and JK curated data, and formal analysis was executed by J-ML, SP, and JiL. JK visualized the results. JiL, JuL, and J-ML wrote the original draft. JiL, JuL, and MK reviewed and edited it. All authors contributed to the article and approved the submitted version.

## Funding

This work was supported by the National Research Foundation (NRF) of Korea, funded by the Ministry of Science, ICT, and Future Planning to JiL (grant number NRF-2019R1A2C1010904). It was also supported by Eulji University in 2022 (JiL). The funder did not have any role in the study design, data collection, analysis, interpretation of data, report writing, and decision to submit the paper for publication.

## Conflict of interest

The authors declare that the research was conducted in the absence of any commercial or financial relationships that could be construed as a potential conflict of interest.

## Publisher’s note

All claims expressed in this article are solely those of the authors and do not necessarily represent those of their affiliated organizations, or those of the publisher, the editors and the reviewers. Any product that may be evaluated in this article, or claim that may be made by its manufacturer, is not guaranteed or endorsed by the publisher.

## Abbreviations

BD: bipolar disorders
BPD: borderline personality disorder
DD: depressive disorders
HR: hazard ratio
NI: non-psychiatric inpatient care
NO: non-psychiatric outpatient care
OAD: other affective disorders
PI: psychiatric inpatient care
PO: psychiatric outpatient care
PTSD: post-traumatic stress disorder
SZ: schizophrenia
