# Supplementary material for: Increased suicide risk of psychiatric patients following the recent utilization of health care services: results from a nationwide cohort study in South Korea
Source: Front Public Health. 2023 May 30;11:1118135. doi: 10.3389/fpubh.2023.1118135 (PMC10261987; doi:10.3389/fpubh.2023.1118135)
Supplement: Supplementary file 1 [file Table_1.docx]

Supplementary Material

**Increased suicide risk of psychiatric patients**

**following the recent utilization of health care services: results from a nationwide cohort study in South Korea**

Ju-Mi Lee, Junhee Lee, Jiseun Lim^*^, Soonjoo Park, Myung Ki, Jiwon Kang

*** Correspondence:** Jiseun Lim, MD, PhD: limjiseun@gmail.com

# Supplementary Description

## Supplementary description of the categorization of mental disorder

We categorized mental disorders into 11 groups to count psychiatric comorbidity based on the Korean Standard Classification of Disease (KCD) code; organic, including symptomatic, mental disorders (F01–F09), mental and behavioural disorders due to psychoactive substance use (F10–F19), schizophrenia, schizotypal, delusional disorders (F20–F29), mood (affective) disorders (F30–F39), neurotic, stress-related and somatoform disorders (F40–F48), behavioural syndromes associated with physiological disturbances and physical factors (F50–F59), disorders of adult personality and behaviour (F60–F69), mental retardation (F70–F79), disorders of psychological development (F80–F89), behavioural and emotional disorders with onset usually occurring in childhood and adolescence (F90–F98), and unspecified mental disorder (F99).

## Supplementary description of time-dependent variables and the time-dependent Cox model

The period from the first diagnosis to the follow-up endpoint was divided into the monthly interval. Then patients' health care utilization (HCU) including psychiatric inpatient care (PI), psychiatric outpatient care (PO), non-psychiatric inpatient care (NI), non-psychiatric outpatient care (NO), and any kind of HCU due to suicide attempts (SA), during each period was investigated (Figure S1).


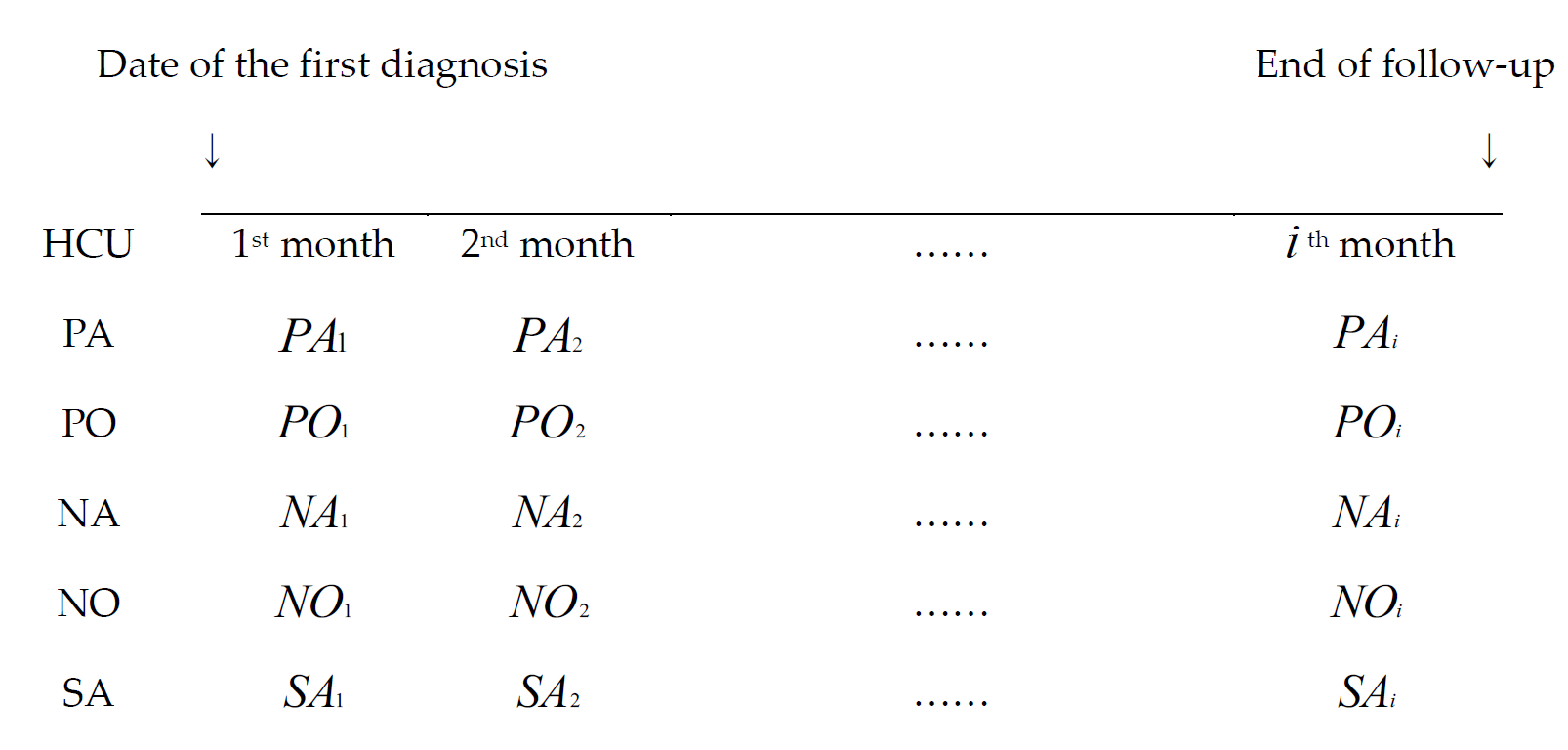


Figure S1. Denotement of health care service utilization at each month



If an individual had HCU (PI, PO, NI, NO, and SA) in $i$^th^ month, then HCU_i_=1, otherwise HCU_i_=0. Assuming that a suicide occurred in the k^th^ month, HCU in the previous month and within the previous six months were calculated as follows.

1. HCU within the previous month:

$PI_{k-1}$,$PO_{k-1}$, $\mathrm{NI}_{k-1}$, $NO_{k-1}$, $\mathrm{SA}_{k-1}$

1. HCU within the previous six months:

, , , ,

Then, these variables were included in the Cox model as follows:

($h(i)$: individual suicide hazard at time $i$, : baseline suicide hazard at time $i$,

: coefficients of HCUs, $PI(i)$, $PO(i)$, $NI(i)$, $NO(i)$, $SA(i)$: HCU at time $i$,

: vector of coefficients of covariates, $C$: vector of covariates)

# Supplementary Table

**Supplementary Table S1.** Adjusted suicide hazard ratio* for recent utilization of health care services, presented in Figure 2

|  | **Schizophrenia** | | | **Bipolar disorders** | | | **Borderline personality disorder** | | | **Depressive disorders** | | | | **Other affective disorders** | | | **Post-traumatic stress disorder** | | |
| --- | --- | --- | --- | --- | --- | --- | --- | --- | --- | --- | --- | --- | --- | --- | --- | --- | --- | --- | --- |
|  | **HR (95% CI)** | | **P** | **HR (95% CI)** | | **P** | **HR (95% CI)** | | **P** | **HR (95% CI)** | | | **P** | **HR (95% CI)** | | **P** | **HR (95% CI)** | | **P** |
| PI in the previous month | 1.90 (1.69-2.14) | <0.001 | | 2.42 (2.10-2.78) | <0.001 | | 1.71 (1.02-2.88) | 0.043 | | 1.43 (1.01-2.02) | 0.043 | | | 3.93 (3.31-4.66) | <0.001 | | 4.67 (2.51-8.69) | <0.001 | |
| PO in the previous month | 2.04 (1.87-2.22) | <0.001 | | 2.46 (2.24-2.69) | <0.001 | | 3.12 (2.29-4.26) | <0.001 | | 1.43 (1.21-1.69) | <0.001 | | | 4.58 (4.24-4.94) | <0.001 | | 4.13 (2.93-5.83) | <0.001 | |
| NI in the previous month | 1.65 (1.43-1.91) | <0.001 | | 1.87 (1.60-2.17) | <0.001 | | 2.07 (1.21-3.53) | 0.008 | | 1.23 (0.86-1.77) | 0.251 | | | 3.29 (2.97-3.65) | <0.001 | | 2.30 (1.33-4.00) | 0.003 | |
| NO in the previous month | 1.14 (1.05-1.24) | 0.002 | | 1.02 (0.93-1.11) | 0.754 | | 1.27 (0.94-1.72) | 0.121 | | 0.64 (0.54-0.77) | <0.001 | | | 1.10 (1.01-1.20) | 0.023 | | 0.90 (0.64-1.27) | 0.535 | |
| PI in the previous six months | 2.34 (2.12-2.58) | <0.001 | | 2.57 (2.30-2.88) | <0.001 | | 1.84 (1.25-2.73) | 0.002 | | 1.78 (1.40-2.27) | <0.001 | | | 3.49 (3.05-4.00) | <0.001 | | 2.95 (1.71-5.09) | <0.001 | |
| PO in the previous six months | 2.96 (2.65-3.30) | <0.001 | | 2.62 (2.34-2.93) | <0.001 | | 3.81 (2.61-5.56) | <0.001 | | 2.04 (1.72-2.42) | <0.001 | | | 4.65 (4.29-5.05) | <0.001 | | 4.70 (3.17-6.96) | <0.001 | |
| NI in the previous six months | 1.55 (1.39-1.74) | <0.001 | | 1.67 (1.50-1.87) | <0.001 | | 1.63 (1.11-2.39) | 0.012 | | 1.28 (1.02-1.62) | 0.037 | | | 2.31 (2.12-2.51) | <0.001 | | 2.48 (1.67-3.69) | <0.001 | |
| NO in the previous six months | 1.03 (0.93-1.14) | 0.589 | | 1.05 (0.93-1.19) | 0.450 | | 0.95 (0.61-1.46) | 0.801 | | 0.62 (0.53-0.74) | | <0.001 | | 0.94 (0.81-1.09) | 0.421 | | 1.04 (0.58-1.85) | 0.897 | | |

(* Suicide hazard ratio after adjustment for age, sex, income, residence area, comorbidity with physical and mental illnesses, and the utilization of other health care services

PI: psychiatric inpatient care, PO: psychiatric outpatient care, NI: non-psychiatric inpatient care, NO: non-psychiatric outpatient care)
